# Microstructure and Mechanical Properties of AZ91 Rein-Forced with High Volume Fraction of Oriented Short Carbon Fibers

**DOI:** 10.3390/ma15144818

**Published:** 2022-07-10

**Authors:** Sabbah Ataya, Mohamed M. El-Sayed Seleman, Fahamsyah H. Latief, Mohamed M. Z. Ahmed, Khalil Hajlaoui, Yousef G. Y. Elshaghoul, Mohamed I. A. Habba

**Affiliations:** 1 Department of Mechanical Engineering, College of Engineering, Imam Mohammad Ibn Saud Islamic University, Riyadh 11432, Saudi Arabia; smataya@imamu.edu.sa (S.A.); fhlatief@imamu.edu.sa (F.H.L.); kmhajlaoui@imamu.edu.sa (K.H.); 2 Department of Metallurgical and Materials Engineering, Faculty of Petroleum and Mining Engineering, Suez University, Suez 43512, Egypt; mohamed.elnagar@suezuniv.edu.eg; 3 Department of Mechanical Engineering, Faculty of Engineering and Science, Universitas Nasional, Jakarta 12520, Indonesia; 4 Mechanical Engineering Department, College of Engineering at Al Kharj, Prince Sattam Bin Abdulaziz University, Al Kharj 16273, Saudi Arabia; 5 Mechanical Engineering Department, Faculty of Engineering, Suez University, Suez 43518, Egypt; yousef.gamal@eng.suezuni.edu.eg; 6 Mechanical Department, Faculty of Technology and Education, Suez University, Suez 43518, Egypt; mohamed.atia@suezuniv.edu.eg

**Keywords:** AZ91 magnesium alloy, carbon fibers, fiber orientation, squeeze casting, composite, microstructure, mechanical properties

## Abstract

In this study, AZ91/23 vol.% short carbon fiber composite was produced by a squeeze casting technique using a cylindrical pre-form of treated carbon fibers, in which the fibers are randomly oriented in the horizontal plane. Cylindrical specimens (height = 9 mm and diameter = 6 mm) were machined from the as-cast AZ91 matrix and its composite. The full behavior of the produced composite was studied through the test specimens machined in two directions, namely parallel to the reinforced plane (in the radial direction of the cast cylinder) and normal to the reinforced plane (in the axial direction of the cast composite). The microstructures of the produced composite specimens were investigated using SEM equipped with EDS analysis. Density, hardness, compressive, and wear behavior were also investigated. For comparison, the AZ91 matrix was evaluated as a reference. The microstructure of the produced AZ91 matrix alloy and its composite revealed dense materials without casting defects. Both composite specimens show improvement in hardness, compressive strength, and wear properties over the AZ91 matrix. The compressive and wear properties are more fiber orientation-dependent than the hardness results. The parallel composite specimen depicts the highest compressive properties in terms of yield compressive strength (311 MPa) and ultimate compressive strength (419 MPa), compared to that shown by the AZ91 matrix and the normal composite specimen. This improvement in compressive strength was at the expense of ductility. The parallel composite specimen shows the lowest ductility (R = 3.8%), compared to that given by the normal composite specimen (R = 7.1) and the AZ91 matrix alloy (R = 13.6). The wear testing results showed that at the highest wear load of 5 N, the material weight loss of the parallel composite specimen decreases by 44% and 64% compared to the AZ91 matrix and the normal composite specimen, respectively.

## 1. Introduction

The issue of weight reduction of mechanical structures has become a popular topic among others in terms of achieving the required CO_2_ emission reduction. The performance of lightweight alloys and their composites has fascinated the automobile and aerospace industries and offers a potential way to substitute existing steel [1]. Thus, scientific research and commercial applications are increasingly focusing on magnesium (Mg) and its alloys due to their low density, which is approximately two-thirds the density of aluminum, high strength-to-weight ratio, and high damping capacity, making them recommended for many engineering applications [2,3]. However, magnesium alloys’ applicability is limited due to their poor creep resistance at high temperatures, low strength, low Young’s modulus, and wear resistance [4,5]. Therefore, more and more engineering applications and industries are turning to metal-matrix composites (MMCs) for their needs [6,7,8].

The MMCs have been produced by incorporating high-strength reinforcing ceramic particles to avoid the drawbacks of their conventional alloys [9]. It is well-known that the properties of the composites are influenced by the size, type (particle or fiber), concentrations, and properties of the matrix and reinforcement [10,11]. Many investigations exhibited that the mechanical properties and stability of Mg alloys at elevated temperatures can be improved by adding reinforcement materials to produce Mg-based composites [12,13]. The most common ceramic particle reinforcement utilized for Mg-based composite strengthening agents are SiC [14], Al_2_O_3_ [15], TiB_2_ [16], etc. Consequently, the addition of ceramic particles generally resulted in a drop in ductility of MMCs, since the nature of ceramic particles is brittle and non-deformable but with enhanced strength responses [16]. Concerning the fibers’ reinforcement, carbon [5], and Al_2_O_3_ [17] have been included in Mg alloy to produce composites with better mechanical performance. Additionally, Mg alloys strengthened with reinforcements in particulates or fibers with micro or nano sizes led to significant improvements in high-temperature strength [18] and creep behavior [4,19]. The dispersion and orientation of the reinforcements have a considerable effect on the mechanical properties of Mg-based MMC. Fibers are the essential type of reinforcements because they have the greatest influence on the directional strength and stability of composites [20]. The addition of carbon fiber is believed to be an effective method for improving the mechanical properties of Mg alloys. Carbon fiber is eventually made of carbonaceous material with a major component of more than 90 wt.% of carbon in a fiber shape, and has outstanding mechanical properties and chemical stability [21].

Moreover, short carbon fiber has been added to Mg-based composites, resulting in a promising material for broader engineering applications [21]. Ataya and El-Magd [5] found that AE42 and AZ91 reinforced with short carbon fiber had at least 2.5 times greater compressive yield strength than their corresponding matrices. Xu et al. [21] investigated that the strengthening effects of short carbon fibers on the AZ31 composite were initially increased and then decreased with increasing short carbon fibers content. However, the low wettability between carbon fibers and Mg alloy as matrix becomes an important issue that should be considered, because the carbon fibers might react with some metallic elements during composite manufacturing. Thus, the adhesion bonding between matrix and fiber would be a critical factor in the determination of the composites’ performance [20,22]. Some efforts have been made to improve the wettability of carbon fibers and Mg by adding alloying elements to control the chemical reaction at the interface, and to optimize the bonding strength between carbon fiber and Mg alloys [23]. The interface design between the Mg alloy matrix and the reinforced carbon fiber, carbon orientation, its volume fraction, and the produced techniques are considered critical parameters for producing Mg-based composites which have a homogenous dispersion of the reinforcement with enhanced properties in the normal and parallel directions of the reinforced plane. At the same time, these processing parameters are a hot topics for researchers, due to their developing uses in the aerospace and transportation industries.

Based on the available literature review, there has been no attempt to deeply study the microstructure and mechanical properties of AZ91-Mg alloy-based composite reinforced with a high-volume fraction of short carbon fiber (23 vol.%) produced via the squeeze casting technique. The physical and mechanical properties of the produced composite were evaluated parallel and normal to the reinforced plane, and then compared with the AZ91 matrix.

## 2. Methodology

### 2.1. Materials

**Figure 1:**
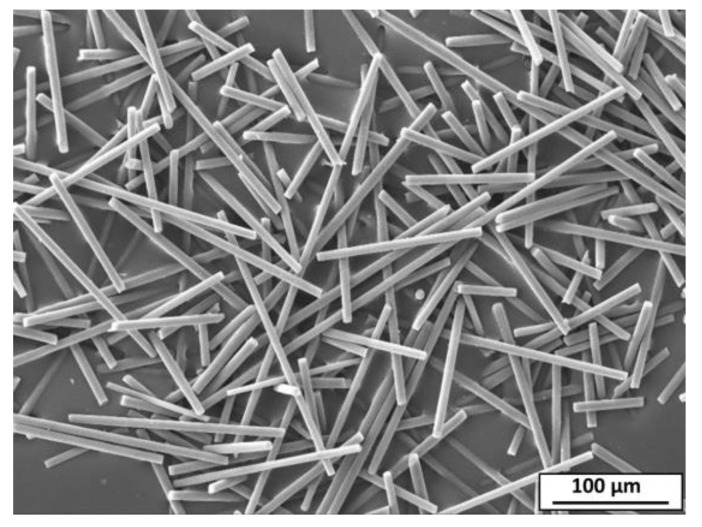
The SEM image of the short carbon fibers.

In the industry, AZ91 magnesium alloy is used to produce the majority of Mg cast parts due to its good mechanical properties and castability. The chemical composition of the AZ91 matrix alloy is listed in Table 1. The short carbon fibers (supplied by SIGRAFIL, SGL Carbon GmbH, Wiesbaden, Germany) with diameter and length ranges of 5–6 µm and 80–120 µm, respectively, are used as reinforcement. Figure 1 illustrates the SEM image of the used short carbon fibers in the current study. Surface treatment of carbon fiber using water silicon was applied to enhance the wettability between the AZ91 and carbon fiber. According to the supplier, the chemical composition and the physical and mechanical properties of the short carbon fibers are given in Table 2.

### 2.2. Production of AZ91/Carbon Fiber Composite

**Figure 2:**
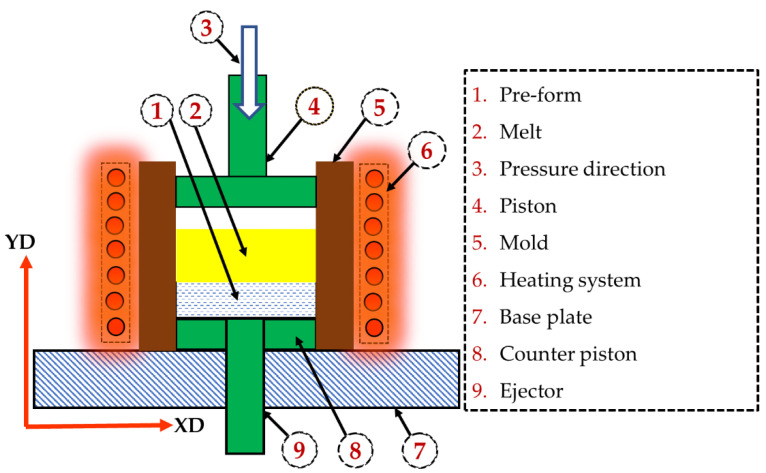
Schematic drawing of the squeeze casting process used in this work, with all items numbered and identified.

The AZ91/short carbon fiber composite is produced with a squeeze casting technique (Figure 2), using AZ91 alloy reinforced with 23 vol.% short carbon fibers. In the squeeze casting process, a pre-form was initially made, and the reinforcement fiber was distributed randomly in the horizontal plane of the pre-form. This pre-form and the casting mold were preheated to 400 °C. The AZ91 melt material was superheated to 730 °C and then poured on that pre-form. Subsequently, a compaction pressure of 80 MPa was applied to flow the AZ91 melt through the short carbon fiber. The squeeze cast composite was solidified at a rapid cooling rate, reaching around 28.2 °Cs^−1^. Additionally, AZ91 without short carbon fiber was produced using the same casting process and conditions for comparison purposes.

### 2.3. Characterization of the Produced Composite

**Figure 3:**
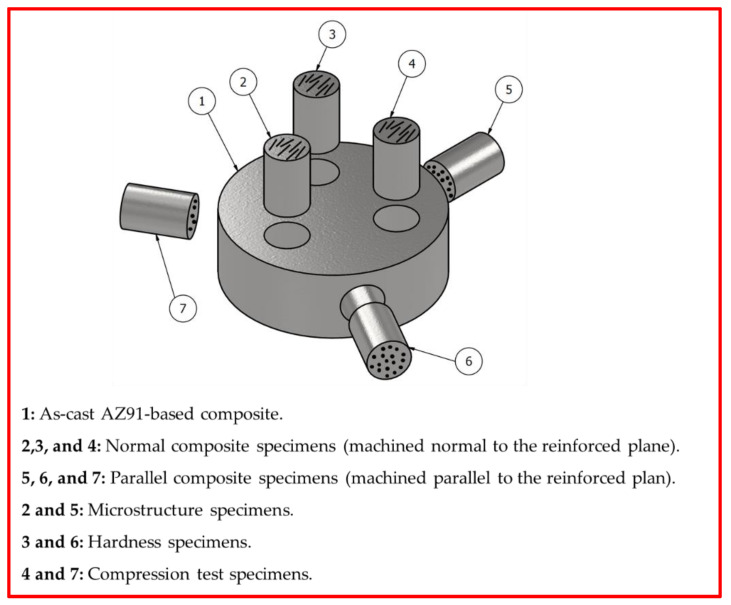
3D drawing shows the machine directions of the test specimens (as-cast AZ91/23 vol.% short carbon fiber).

Cylindrical specimens of the as-cast materials in dimensions of 9 mm in length and 6 mm in diameter were machined for different testing and investigations. To study the full behavior of the produced materials, the AZ91/short carbon fiber composite specimens were machined in two directions, as follows: firstly, samples were machined parallel to the squeeze pressure direction (axial direction), which is perpendicular to the reinforced plane, and secondly, the samples were machined perpendicular to the pressure direction (radial direction) which is parallel to the reinforced plane, as shown in Figure 3. The density of the as-cast AZ91 matrix and composite specimens were measured using Archimedes’ principle. The theoretical density was estimated using the law of mixture, and the relative densities were calculated. Hardness measurements were obtained with a Vickers hardness tester (Model: HWDV-7S, TTS Unlimited, Osaka, Japan) using a 20 N load and a 15 s holding time. The average hardness value was taken as an average of eight measurements for each specimen. Compression tests were carried out using a universal testing machine (Model: Schenck-Trebel RMC100, Deer Park, NY, USA) with a 0.001 s^−1^ strain rate at room temperature. Microstructure investigation has been conducted for the as-cast AZ91 matrix alloy and the AZ91/23 vol.% composite materials using a scanning electron microscope (SEM, Quanta FEG 250, Hillsboro, OR, USA), equipped with an energy-dispersive X-ray spectrometry (EDS) analysis system (TEAM EDS Analysis, version 3.3, AMETEK, Heerenberg, Netherlands). A comparative weight loss wear test under the drying condition was carried out for all the produced specimens (AZ91 matrix and the AZ91/23 vol.% short carbon fiber composite) using a pin-on-disc machine (Model: WT-M1-SSMMR-CSE, Suez, Egypt) at different applied loads of 2, 3, 4, and 5 N and a constant sliding distance of 2.3 × 10^6^ cm. The worn surface of the tested specimens was also investigated using the SEM/EDS system.

## 3. Results and Discussion

### 3.1. Microstructure Analysis

**Figure 4:**
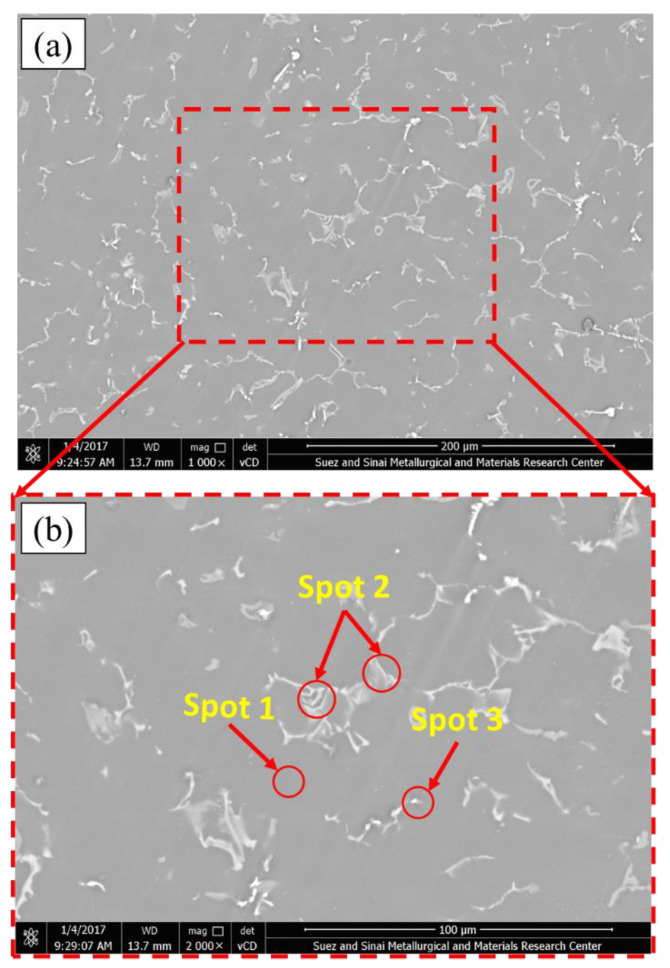
(**a**) Low and (**b**) high magnification SEM images of the as-cast AZ91 microstructure.

**Figure 5:**
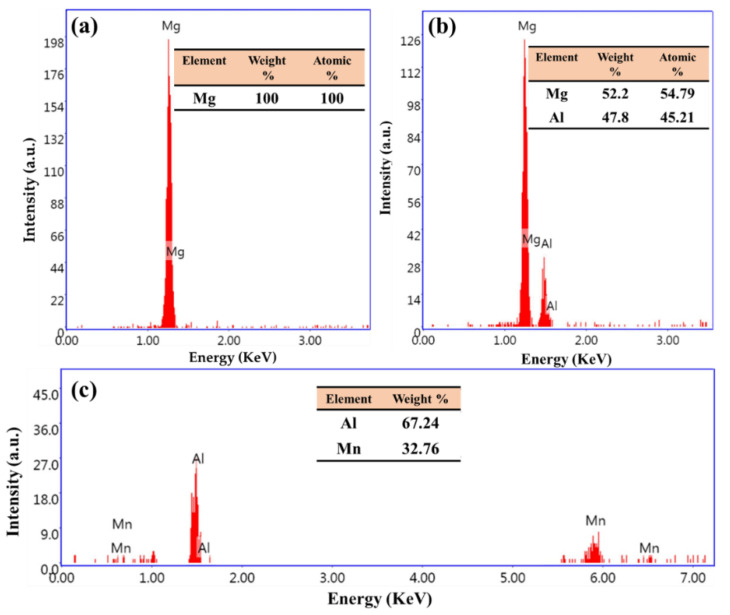
EDS analysis of the AZ91 matrix and the two intermetallics; (**a**) Mg matrix (spot 1 in Figure 4), (**b**) β-Mg_17_Al_12_ (spot 2 in Figure 4), and (**c**) Al_4_Mn (spot 3 in Figure 4).

**Figure 6:**
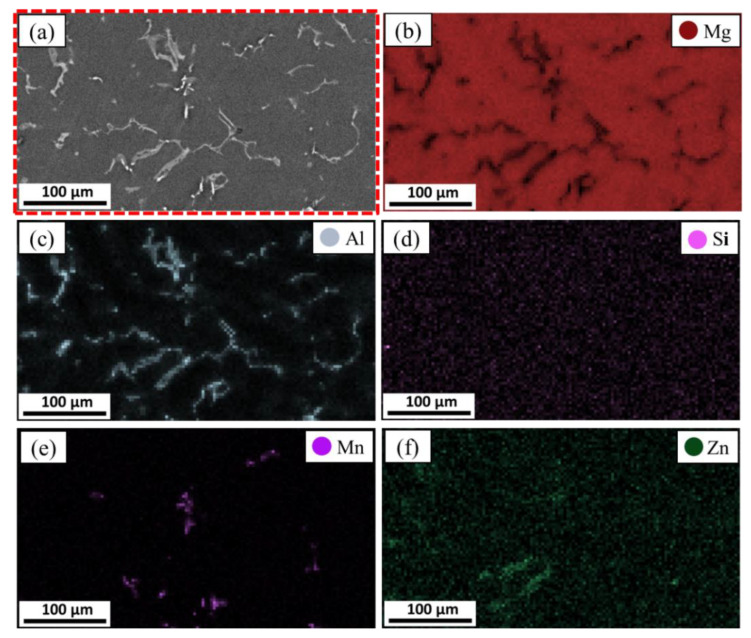
Elemental mapping of the as-cast AZ91 alloy: (**a**) SEM-image shows the selected area microstructure of AZ91 for mapping analysis and the map distribution of main alloying elements: (**b**) Mg, (**c**) Al, (**d**) Si, (**e**) Mn, and (**f**)Zn.

The morphology of the intermetallics in Mg alloys is dependent on various variables, including the casting technique, cooling rate, and alloying elements [24,25,26]. Here, SEM equipped with an advanced EDS analysis system was used to study the microstructure and intermetallics of the as-cast AZ91 matrix alloy and the AZ91/23 vol.% short carbon fiber composite. The AZ91 matrix has the alloying elements of Al, Si, Zn, and Mg, as listed in Table 1, and Figure 4, Figure 5 and Figure 6 show the microstructure (low and high magnifications), EDS analysis, and elemental mapping of the as-cast AZ91 matrix, respectively. It can be noted that the microstructure mainly consists of a large dendritic structure of α-Mg matrix (smooth grey areas) with secondary arms, as shown in Figure 4a. This matrix phase is confirmed by the point EDS analysis (spot 1) in Figure 4b and represented in Figure 5a. Two different intermetallics were also remarked in the as-cast AZ91 microstructure. The first intermetallic is the bright layers at the grain boundaries of the α-Mg matrix, namely eutectic β-Mg_17_Al_12_ and α-Mg lamellar. It is also detected as a bright solid area on the grain boundaries, namely the lamella secondary β-Mg_17_Al_12_ (spot 2 in Figure 4b and given in Figure 5b). This β-Mg_17_Al_12_ + α-Mg intermetallic formed from aluminum supersaturated the α-Mg via discontinuous precipitation (solid-state reaction) [27]. The second detected intermetallic in the α-Mg matrix is Al_4_Mn precipitates (manganese phase), as shown in Figure 4b (spot 3) and represented in Figure 5c. These microstructure features of the as-cast AZ91 are detected by other authors [5,28,29]. The elemental mapping of the different alloying elements of the AZ91 was performed on the microstructure to study the microstructure homogeneity of the produced AZ91 alloy via the applied squeeze casting technique. The elemental mapping (Figure 6) shows the composition and location of the main alloying elements found in the AZ91, namely Mg (Figure 6b), Al (Figure 6c), Si (Figure 6d), Mn (Figure 6e), and Zn (Figure 6f). The elemental mapping results agree well with the chemical composition of AZ91 (Table 1) and confirm the formation and location of the intermetallics throughout the whole material, indicating the structural homogeneity of the typical microstructure of the as-cast AZ91 [29,30].

**Figure 7:**
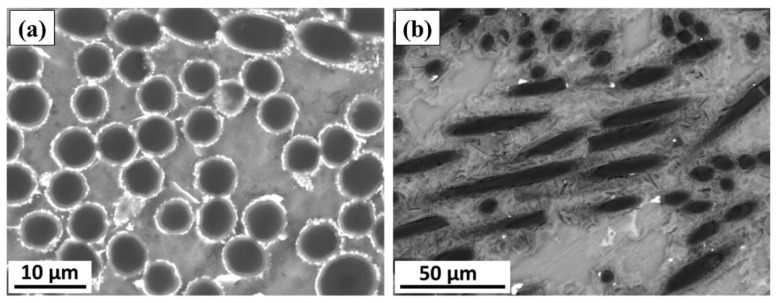
SEM images showing the microstructure of the AZ91/23 vol.% carbon fiber composite specimens machined; (**a**) parallel and (**b**) normal to the reinforced plane.

The dispersion of the short carbon fibers which reinforced the AZ91 matrix alloy to produce AZ91/23% carbon fiber composite was studied for the specimens machined in the parallel and normal directions to the reinforced plane (Figure 3) using SEM. Figure 7 shows the SEM images of the parallel (Figure 7a) and normal (Figure 7b) reinforced composite specimens. It can be seen that the short fibers dispersed separately and bonded well with the AZ91 matrix in both cases, without being in overlapping or intersecting bundles. The circular cross-sections of the short carbon fibers in Figure 7a are observed in the range of the initial cross-section diameter of the used carbon fiber (Figure 1). Furthermore, there is no porosity or casting voids remarked upon in the AZ91 matrix (Figure 4) and the AZ91/23 vol.% carbon fiber composite specimens in both directions (Figure 7), indicating the role of the high pressure used during the solidification process of the squeeze cast materials, via the suggested producing parameters in the current study.

**Figure 8:**
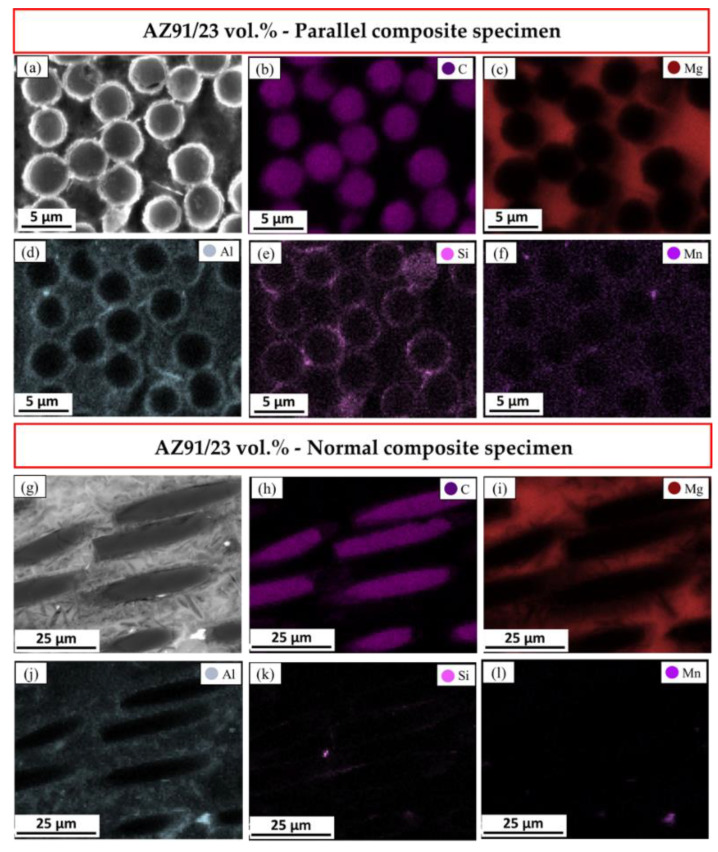
Elemental mapping of AZ91/23 vol.% carbon fiber for the (**a**–**f**) parallel and (**g**–**l**) normal composite specimens.

To investigate the dispersion and location of the AZ91 alloying elements in the presence of the reinforced 23 vol.% short carbon fibers, the elemental mappings are carried out on the microstructure of the produced composite specimens. This is presented in Figure 8a–f for the parallel and in Figure 8g–l for the normal composite specimens. It can be exhibited that the elemental maps in Figure 8 confirm the alloying elements of the AZ91 matrix in addition to the short carbon fiber coated with Si. The Si element, which resembles concentrated dots, appear as bright areas in the SEM image (Figure 8a) around the cross-section of the carbon fibers. The elemental Si maps in Figure 8e,k represent the location of this element as a coating on the surface of the carbon fibers for the parallel and normal composite specimens, respectively. It can be noted that the Si concentration (the coating layer of the carbon fiber) is highly observed in the microstructure of the parallel composite specimen compared to the normal specimen. The loss in the detection of Si-coated fiber in the longitudinal direction of the reinforced carbon fiber of the SEM investigated normal composite specimen may be ascribed to the action of the grinding and polishing processes. The chemical reaction between aluminum and carbon at high temperatures leads to the formation of carbides. This phenomenon cannot be avoided at the interface between the Mg matrix, as the interface contains a certain aluminum concentration and the uncoated reinforcing carbon phase, which dramatically reduces the produced composite’s strength [12]. The elemental mapping of the produced composite revealed that there is almost no aluminum in direct contact at the interface of the carbon fibers. This indicates that the coated Si layer around the carbon fiber improves the wettability of the fiber with the Mg matrix and prevents the formation of aluminum carbide. It can be concluded that the AZ91/23 vol.% carbon fiber composite produced by the squeeze casting technique effectually restrains harmful interfacial reaction and avoids producing a brittle phase, which is adverse to the properties of the produced composite.

### 3.2. Physical and Mechanical Properties

Table 3 lists the density and hardness properties of the as-cast AZ91 matrix and the two AZ91/23 vol.% carbon fiber composite specimens. It is observed that there is no remarked difference in the relative density values between the as-cast AZ91 and the two composite specimens (Table 3). This indicates the successful applied squeeze casting technique (using high pressure during the solidification process) in producing dense Mg-cast materials with and without reinforced short carbon fibers at a high-volume fraction of 23%. The density results agree well with the remarked microstructure features for the as-cast AZ91 (Figure 4) and the AZ91/23 vol.% carbon fiber composites (Figure 7a,b). Significant improvement in hardness of the parallel and normal composite specimens is noted over the AZ91 matrix, as listed in Table 3. The hardness of the AZ91 matrix alloy is 70 ± 3 HV. This value increased for the produced AZ91/23 vol.% short carbon fiber composite to 111 ± 2 HV and 106 ± 4 HV for the parallel and normal composite specimens, respectively. This enhancement in hardness reveals the reinforcing effectiveness of the high-volume fraction of short carbon fibers to the AZ91 matrix. Many works showed that the hardness of the Mg alloys improves with the addition of short carbon fibers to produce composites [2,31,32]. Ajukumar et al. [31] reported around 16% enhancement in hardness for the AZ91 matrix reinforced with only 2.5 wt.% short carbon fibers. In addition, Myalska and Myalski [32] reported a hardness increment of 22% of the AZ31 matrix with the addition of 7 vol.% short carbon fibers. Ataya et al. [2] concluded that reinforcing AE42 Mg alloy with 23 vol.% random short carbon fibers increases the hardness by 100%. The hardness test causes localized deformation (elastic and plastic) under the indenter. The yielded indentation is governed by the elastic modulus of the tested material (load carrying capacity). The load carrying capacity of the AZ91/23 vol.% carbon fiber composite is higher than that given by the AZ91 matrix, where the elastic modulus of the short carbon fiber (280 GPa) is higher than the AZ91 matrix (44.8 GPa). The presence of higher elastic modulus reinforcements compared to the metal matrix causes inhomogeneous deformation and leads to an increase in dislocation density. Turan et al. [11] reported that the hardness performance of AZ91 is improved significantly with the addition of carbonaceous reinforcements, and ascribed that this enhancement may be due to the hindrance of the dislocation movement by the carbon-based reinforcements.

**Figure 9:**
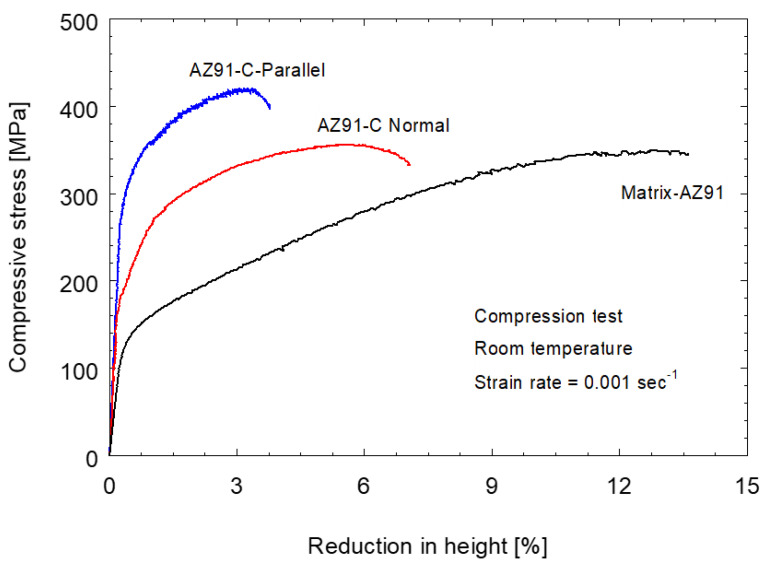
The compressive stress against the reduction in the height for the AZ91 matrix and AZ91/23 vol.% carbon fiber composite specimens.

Since the Mg-based composites are primarily used as piston materials in applications involving significant compression loads, it is essential to study the behavior of these materials under compressive stresses. Figure 9 shows the compressive stress–strain curves for the as-cast AZ91 alloy and AZ91/23 vol.% carbon fiber composite samples machined to be loaded parallel and normal to the reinforced plane. It can be remarked that the addition of 23 vol.% carbon fibers to the AZ91 matrix to produce a composite increases both yield compressive strength (YCS) and ultimate compressive strength (UCS) and decreases the reduction in height (R) of the tested specimens compared to the AZ91 base alloy. The improvement in the UCS and YCS of the composite materials over the matrix alloy is ascribed to the high modulus of elasticity of the carbon fibers compared to the AZ91 matrix alloy and the primary acting mechanism is likely to be the fiber reinforcing mechanism. The compression test causes bulk deformation for the tested material. The load carrying capacity of the tested material, which is correlated with the elastic modulus, controls how much deformation occurs. The load carrying capacity of the AZ91/23 vol.% carbon fiber composite is higher than that recorded for the AZ91 matrix, where the elastic modulus of the short carbon fiber is higher than that of the AZ91 matrix. Thus, the composite materials possess higher yield and compressive strengths than those recorded with the AZ91 matrix. Many researchers [11,33,34] reported that the introduction of carbon-based reinforcements result in an improvement in the strength of the produced metal matrix composites. This positive effect on the strength may be due to the dislocation movement effected by carbon-based reinforcement, which results in the load transfer effect mechanism.

Table 4 summarizes the compression test results in the UCS, YCS, and R of the AZ91 and the two tested composite specimens. It can be noted that the parallel composite specimen gives the highest UCS (419 MPa) and YCS (311 MPa) compared to the normal composite specimen and the AZ91 matrix alloy. This improvement in compressive strength was at the expense of ductility. The parallel composite specimen shows the lowest ductility (R = 3.8%) than that given by the normal composite specimen and the AZ91 matrix alloy. A similar finding of the fiber orientation effect on the compression testing behavior has been noticed in testing the Mg-alloy AE42 and its composites (AE42/23 vol.% carbon fiber) [5]. There is a significant contribution of the carbon fibers in the load carrying capacity of the composite loaded parallel to the reinforced fibers (the parallel composite specimen, Table 4). This is due to the longitudinal fibers directly absorbing, transferring, and distributing the applied load uniformly throughout the cross-section of the tested material [35,36,37]. On the other hand, when loading the AZ91/carbon fiber composite normal to the reinforced fibers the contribution of the matrix seems more significant in the increased ductility and lower compressive strength.

**Figure 10:**
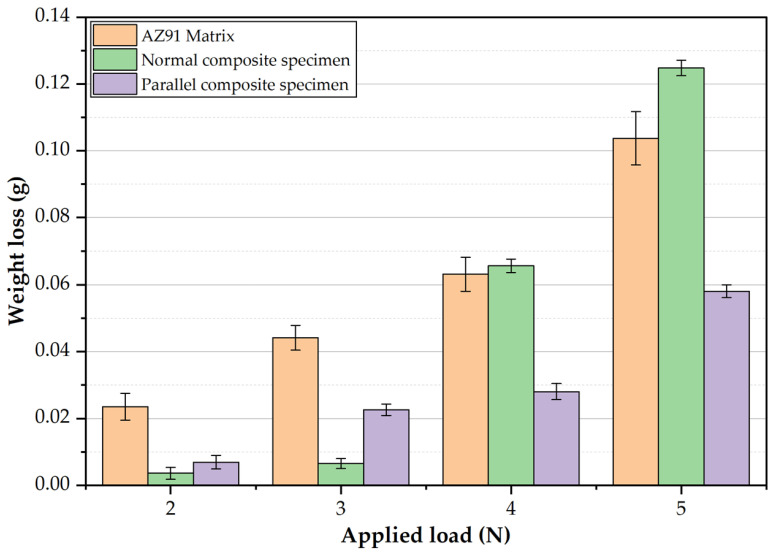
Weight loss of the AZ91 matrix and AZ91/23 vol.% short carbon fiber composite specimens, conducted at different loads and a constant sliding distance of 1.34 × 10^4^ m.

The wear behavior of the AZ91 matrix and the AZ91/23 vol.% short carbon fiber composite specimens were evaluated in terms of weight loss at wear test conditions of different applied loads (2, 3, 4, and 5 N) and a constant sliding distance of 2.3 × 10^6^ cm, as shown in Figure 10. It can be seen that the weight loss of the AZ91 matrix and the two composite specimens increases when increasing the applied wear load from 2 to 5 N. It can also be observed that there is a significant improvement in the wear resistance of the composite materials (parallel and normal specimens) over the base Mg matrix alloy at the applied wear loads of 2 and 3 N. At the lowest applied wear load of 2 N, the material weight loss of the parallel composite specimen (carbon fibers parallel to the sliding direction) and the normal composite specimen (carbon fibers perpendicular to the sliding direction) decrease by 77% and 85%, respectively, compared to the AZ91 matrix. Whereas, at the applied wear loads of 4 and 5 N, the parallel composite specimen shows a significant enhancement in the wear resistance compared to the AZ91 matrix and normal composite specimen, respectively. This improvement is ascribed to the action of the formation of the carbon lubricant film between the rubbing surfaces (the composite material and the rotating wear disk). This reduces the level of component shear, leading to less metal removal, and finally decreases the weight loss [2,38]. It should be noted that the carbon fiber orientation influences the weight loss of the AZ91 composite specimens.

At the highest wear load of 5 N, the material weight loss of the parallel composite specimen decreased by 44% and 64% compared to the AZ91 matrix and the normal composite specimen, respectively. In the parallel composite specimen, the carbon fibers are normal to the sliding direction. This promotes a continuous feeding of carbon lubricant film between the rubbing surfaces, which effectively reduces frictional heat and weight loss compared to the discontinuous film feeding in the normal composite specimen (the carbon is parallel to the sliding direction). At the wear loads of 4 and 5 N, the applied shear force on the worn surface of the normal composite is higher than the cohesion force between the carbon fibers and the AZ91 matrix. This causes carbon fiber pullout due to the presence of the interfacial deboning as a result of thermal expansion mismatch between the carbon fibers and the AZ91 matrix. The axial thermal expansion coefficient of carbon fiber is 1.48 × 10^−6^ K^−1^, while the thermal expansion coefficient of AZ91 alloy is 26.8 × 10^−6^ K^−1^ [39]. The pullout of carbon fibers causes material loss without utilizing the inherent lubricity property. This phenomenon is detected only with the normal composite and combined with delamination layers of AZ91 matrix, due to the accumulated friction heat and severe plastic deformation. These two reasons cause deterioration in the wear properties in terms of weight loss of the normal composite specimen compared to the parallel composite and the AZ91 matrix at the highest applied wear load of 5 N. Qi et al. [20] reported a decrease in the weight loss of the AZ91/10 vol.% short carbon fiber composites compared to the AZ91 matrix alloy. They also concluded that the fiber orientation governs the wear behavior of the composite.

**Figure 11:**
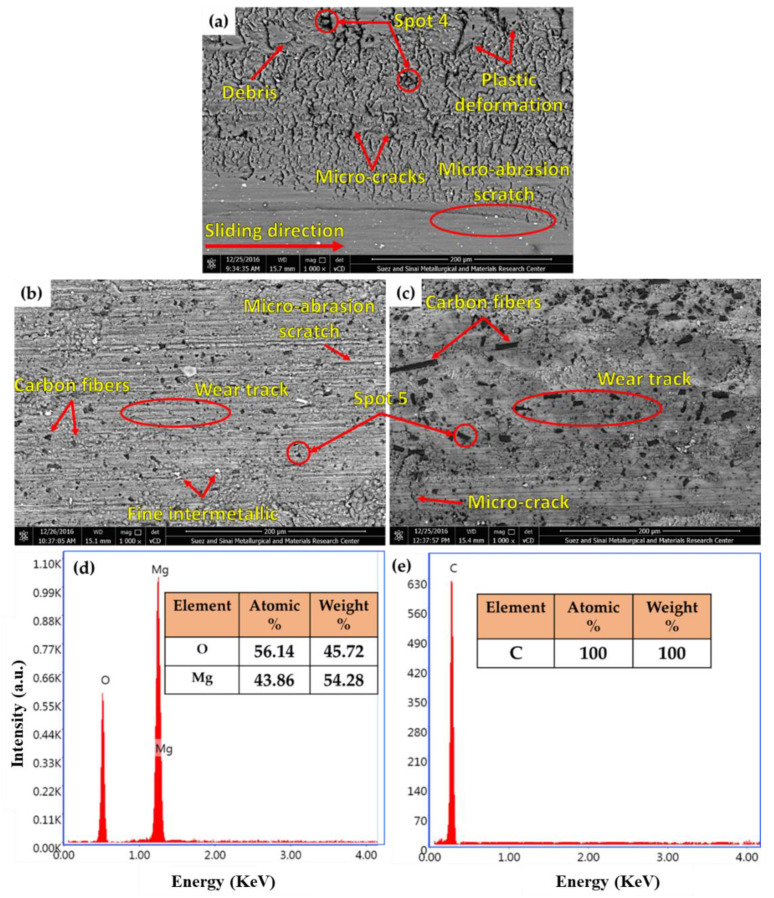
Worn surface SEM images of the (**a**) AZ91 matrix, (**b**) parallel composite, and (**c**) normal composite specimens at the applied wear load of 5 N, and a constant sliding distance of 1.34 × 10^4^ m. Additionally, the EDS spot analysis of (**d**) MgO; (spot 4 in (**a**),(**e**)) carbon fibers; C (spot 5 in (**b**,**c**)) is shown.

Figure 11 illustrates the worn surface of the AZ91 matrix, parallel, and normal composite specimens at the applied wear load of 5 N, and a constant sliding distance of 1.34 × 104 m. It can be seen that the worn surface features of the AZ91 matrix are transverse microcracks, continuous scratches parallel to the sliding direction, plastic deformation, partial oxidation (Spot 4 in Figure 11a and represented in Figure 11d), and delamination layers, indicating that the wear mechanisms are plastic deformation, delamination layer, and abrasion [2]. The plastic deformation and the delamination layers are ascribed to the accumulative friction heat between the rubbing surfaces (the AZ91 matrix and hard steel). Additionally, the abrasion wear may ascribe to the free ceramic particles (intermetallic fragmentation and MgO formation) between the two rubbing surfaces. In a deep study, Aydin and Durgut [40], examined the wear behavior of the AZ91 alloy under different wear conditions. They found that the volume loss of the AZ91 alloy increased when increasing the applied loads from 10 to 50 N for all the sliding distances (250–1000 m). In addition, the wear mechanisms are abrasion and oxidation at the lowest load of 10 N, which changes into a delamination mechanism at the highest load of 50 N.

The worn surface of the wear-tested composite specimens reveals that the main features are the shallow wear track and lower micro-cracks compared to that given with the AZ91 matrix. In addition, the smearing of graphite film (dark gray areas in Figure 11b,c) on the worn surface in both composites and abrasion scratch are also observed. Some carbon fibers (spot 5 in Figure 11a and represented in Figure 11e) may experience pullout in the case of the normal composite specimen (carbon fibers parallel to the sliding direction suffer from friction shear force). These features indicate that the wear mechanisms are abrasion and delamination mechanisms [2,20]. In fact, the amount of the smearing film of the graphite solid lubricant governs the wear performance of the AZ91 self-lubricating composite materials against the harder rubbing counterface. This leads to a reduction in material weight loss. Aydin and Turan [41] reported that the wear resistance of the AZ91 Mg-based composite containing nano boron nitride was enhanced over the Mg base material at different wear conditions, due to the action of the solid lubricant boron nitride on the composite worn surface. They reported that the wear mechanisms are thermal softening, melting, oxidation, abrasion, and delamination.

## 4. Conclusions

Based on the analysis of the results, the following conclusions can be outlined:

1. The squeeze casting process successfully produced a highly dense AZ91-based composite containing a high-volume fraction of carbon short fibers (23%), which bonded well with the matrix and highly distributed in the normal and parallel direction to the reinforcing plane.
2. Reinforcing AZ91 with 23 vol.% short carbon fibers increases the hardness of the AZ91 matrix by not less than 51% for the parallel and normal composite specimens.
3. The yield compressive strength and the ultimate compressive strength of the composite are higher than those recorded for the AZ91 matrix. Moreover, the parallel fiber composite specimen gives the highest yield compressive strength (311 MPa) and ultimate compressive strength (419 MPa), compared to the normal fiber composite specimen and the AZ91 matrix alloy.
4. At the constant sliding distance of 2.3 × 10^6^ cm and applied wear loads of 2 and 3 N, the composite specimens (parallel and normal specimens) show a significant improvement in wear resistance over the AZ91 matrix alloy. Additionally, at the wear loads of 4 and 5 N, the parallel composite specimen shows a remarked enhancement in wear resistance compared to the AZ91 matrix and the normal composite specimen.

## Figures and Tables

**Table 1:** The chemical composition of the AZ91 Mg alloy.

Element	Mn	Si	Zn	Al	Mg
wt.%	0.13	0.05	1.00	9.00	Bal.

**Table 2:** The chemical composition and mechanical properties of short carbon fibers.

Chemical Composition	Tensile Strength	Elastic Modulus	Density
>95 wt.% C	3.5 GPa	280 GPa	1.76 g/cm^3^

**Table 3:** Measured, theoretical, and relative density and hardness of the casted materials; the AZ91 matrix and the AZ91/23 vol.% carbon fiber composite specimens.

Material	Bulk Density g/cm^3^	Theoretical Density g/cm^3^	Relative Density (%)	Hardness (HV)
**AZ91 as-cast alloy**	1.8080 ± 0.003	1.8060	0.9989	70 ± 3
**Parallel composite specimen**	1.7950 ± 0.004	1.7768	0.9899	111 ± 2
**Normal composite specimen**	1.7800 ± 0.008	1.7602	0.9889	106 ± 4

**Table 4:** Summarizes the ultimate compressive strength (UCS), yield compressive strength (YCS), and reduction in height (R) values for the AZ91 matrix and AZ91/23 vol.% carbon fiber composite specimens.

Material	UCS (MPa)	YCS (MPa)	R (%)
**AZ91 as-cast alloy**	348 ± 9	139 ± 7	13.6 ± 1.2
**Parallel composite specimen**	419 ± 15	311 ± 12	3.8 ± 0.6
**Normal composite specimen**	356 ± 13	192 ± 10	7.1 ± 0.8

## Data Availability

The data presented in this study are available on request from the corresponding author. The data are not publicly available due to the extremely large size.
